# Free Radical Scavenging Effect and Immunomodulatory Activity of Total Saponins Extract of Ginseng Fibrous Roots

**DOI:** 10.3390/molecules29122770

**Published:** 2024-06-11

**Authors:** Peng Zhang, Dongyan Zhang, Chuanjie Ma, Ruxia Wang, Weili Wang

**Affiliations:** 1 College of Life Engineering, Shenyang Institute of Technology, Fushun 113122, China; z182313@163.com (D.Z.); tj15710554320@163.com (C.M.); myxydw2021@163.com (R.W.); 2 Liaoning Inspection, Examination & Certification Centre, Shenyang 110031, China

**Keywords:** ginseng fibrous roots, total saponin, extraction process, chemical constituents, antioxidant capacity, immunomodulatory activity

## Abstract

Ginseng (Panax ginseng C.A. Mey) is known for its rich saponin compounds and tonic effects. To better utilize the medicinal value of ginseng, this study investigated the extraction process, components, free radical scavenging ability, and immunomodulatory activity of total saponins of ginseng fibrous roots. The response surface methodology was employed to optimize the extraction process of total saponins, and Q-Orbitrap high-resolution liquid chromatography–mass spectrometry (LC-MS) was used to identify the chemical constituents in the total saponins extract of ginseng fibrous roots (GRS). The results showed that the optimal extraction process was achieved with an ethanol concentration of 68%, a material–solvent ratio of 1:25 mL/g, and an extraction time of 20 min, yielding a total saponin content of 6.34% under these conditions. The extract contained four terpenoid compounds and four polyphenolic compounds. GRS exhibited considerable scavenging activity against DPPH and ABTS radicals, with IC_50_ values of 0.893 and 0.210 mg/mL, respectively. Moreover, GRS restored immune suppression in mice by increasing white blood cell, red blood cell, and neutrophil counts, and improving the lymphocyte. It also promoted immune system recovery, as evidenced by elevated serum levels of IL-2, IFN-γ, TNF-α, and IL-1β in mice. GRS is a natural compound with promising potential for developing antioxidants and immunomodulatory foods.

## 1. Introduction

It is widely recognized that the body’s immune system serves as a defense network, with roles in immune surveillance, defense, and regulation, effectively preventing pathogen invasion and efficiently recognizing and eliminating foreign antigens to maintain the relative stability of the body’s internal environment [1,2]. As a safety mechanism, its normal operation holds significant importance for bodily functions. With the continuous advancement in herbal medicine research, scholars have gradually recognized the vast prospects of active ingredients in plants for regulating the body’s immune system. On one hand, herbal medicines can enhance the cellular and humoral immune functions of the body, promoting the physiological functions of lymphocytes, mononuclear macrophages, and hematopoietic stem cells [3,4,5]; on the other hand, they also possess immune-suppressive functions, reducing the release of inflammatory factors, inhibiting or eliminating antibody production, suppressing T cell proliferation, and countering oxidative stress [6,7,8]. Current research has found that the majority of herbal medicines have bidirectional immune-regulating functions, restoring excessive or deficient immune responses to normal levels. This bidirectional immune modulation embodies the holistic view and the theory of Yin–Yang balance emphasized in traditional Chinese medicine.

Excessive free radicals have been shown to damage the structure and function of cells, cause immune system disorders, accelerate aging, and induce a variety of diseases. Ginseng acts as a natural antioxidant, with active ingredients abundant in ginseng polysaccharides, saponins, and volatile oils playing an active role in antioxidants and oxygen-free radical removal. Luo et al. [9] obtained two neutral polysaccharides, GPII and GPIII, with robust antioxidant activity, through the process of boiling water extraction and the purification of ginseng. The results showed that both GPII and GPIII have slightly higher scavenging abilities for hydroxyl radicals and superoxide radicals than VC. Saw et al. [10], by studying the potential activation and synergistic effects of ginseng saponin Rb1, Rg1, and ginsenoside 20(S)-protopanaxatriol (20S) on Nrf2-ARE-mediated transcriptional activity, found that saponin Rb1, Rg1, and 20S possess antioxidant activity and synergistically activate ARE. Wang et al. [11], in their study on Caenorhabditis elegans, found that the DPPH free radical scavenging rates of ginseng volatile oils at concentrations of 12.5, 25, and 50 μg/mL were 74.67%, 76.3%, and 71.39%, respectively.

Ginseng fibrous roots refer to the dried fine roots or fibrous roots of the Panax ginseng C. A. Mey, a plant belonging to the Araliaceae family. The ancient pharmacopeia *Shennong Bencaojing* (also *Classic of the Materia Medica*) mentions that ginseng has the effects of greatly tonifying the qi, restoring the pulse and securing the collapse, producing fluids and nourishing the blood, calming the spirit and enhancing intelligence, and benefiting the spleen and augmenting the lungs. Modern pharmacological studies have shown that ginseng has various effects, including enhancing the body’s immune system, combating tumors, delaying aging, and alleviating fatigue [12,13,14,15]. Ginseng saponin is a main active ingredient in ginseng, and its content is also the main indicator for evaluating the intrinsic quality of ginseng. Among them, ginseng saponin Rb1, Rb2, Rc, Rd, Rg1, and other saponins account for more than 80% of total ginseng saponin, which is considered to be the main ingredient of ginseng saponin. Studies have shown that RT5, Rh2, oleanolic acid β-D-glucopyranosyl ester [16], Rh1 [17], Rg3 [18], and Rb1 [19] are directly involved in immune regulation. In recent years, ginseng extracts have also been proven to have immunomodulatory activities, including enhancing macrophage phagocytic activity, increasing NO production, and regulating cytokines such as IL-1, IL-6, TNF-α, and IL-12 [20,21,22].

Currently, there is a growing popularity in the utilization of botanical products for treating various diseases. People agree that herbal medicines have unlimited potential and are a relatively healthy treatment option with minimal side effects. With the rapid advancement in the ginseng planting industry, ginseng by-products such as fibrous roots are not fully utilized. In addition, the composition of each ginseng preparation may be affected by factors such as growth temperature, altitude, and weather conditions at different planting sites [23]. Therefore, the purpose of this study is to extract total saponins from ginseng fibrous roots in the Liaodong region, China, to identify its chemical constituents, and to evaluate its ability to remove free radicals and immunomodulate.

## 2. Results and Discussion

### 2.1. Optimization of Single-Factor Experiments

**Figure 1:**
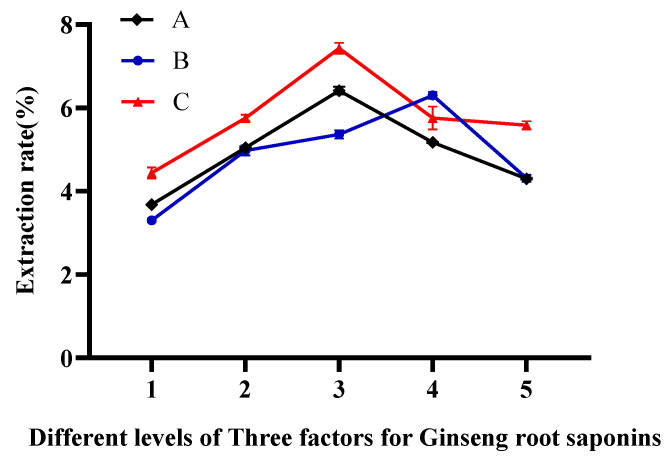
Effect of ethanol solution concentration (A: 50%, 60%, 70%, 80%, 90%), solvent–material ratios (B: 1:30, 1:25, 1:20, 1:15, 1:10 mL/g), and ultrasonic extraction time (C: 10, 15, 20, 25, 30 min) on GRS extraction rate.

Scholars Li et al. [24], in their study of the effect of different extraction methods on the content and structural transformation of ginsenosides, found that the content of ginsenosides gradually increased when 50–70% ethanol was used as the solvent; the extraction time peaked at 30 min, with the total ginsenoside content reaching 25.16 mg/g, after which the content gradually declined; when the ratio of the material to liquid was 1:20 and 1:30, the total ginsenoside content was 33.53 mg/g and 33.50 mg/g, respectively, indicating higher extraction efficiency. Hen et al. [25] utilized an ultrasound-assisted multistage countercurrent extraction technique and determined that the optimal conditions for obtaining the highest ginsenoside content were an ethanol concentration of 70%, a material–liquid ratio of 1:10, and an extraction time of 30 min. Therefore, this experiment was designed with reference to the optimal results from various studies, and the investigated conditions included ethanol concentration (50%, 60%, 70%, 80%, 90%), material–liquid ratios (1:30, 1:25, 1:20, 1:15, 1:10 mL/g), and ultrasonic times (10, 15, 20, 25, 30 min). These conditions were tested to obtain the one-factor highest extraction conditions. The results of the three single-factor experiments are shown in Figure 1. The yield of GRS gradually increased within the range of 50% to 70% ethanol concentration. However, if the ethanol concentration continues to increase beyond this range, the yield of GRS gradually decreases. Therefore, the optimal ethanol concentration for GRS extraction is 70%. With the increase in the material–solvent ratio, the yield of GRS also continuously increases. However, after further increasing the solvent volume, the yield of GRS decreases. Therefore, a material–solvent ratio of 1:25 is the optimal extraction condition. Additionally, the yield of GRS continuously increases from 10 min to 20 min of extraction time. Within the range of 20 min to 30 min, the yield of GRS starts to decline. Hence, 20 min is selected as the optimal extraction time for GRS.

### 2.2. Optimization of Extraction Conditions by BBD

Based on the results of the single-factor experiments in Section 2.1, the three factors at three levels, namely the ethanol solution concentration (60%, 70%, 80%), ultrasonic extraction time (10 min, 20 min, 30 min), and solvent–material ratio (20:1, 25:1, 30:1 mL/g), were applied to the RSM design. The process for extracting total saponins from ginseng fibrous roots was optimized based on the response surface design and the independent variables for the experiment. A total of 17 experimental sets were designed, each varying across three levels. The effect of these variables on the extraction rate of GRS was examined. The experimental design and results are shown in Supplementary Table S1. Through the analysis and integration of experimental data using a regression analysis, the obtained Quadratic multiple regression equation was as follows: Y = 6.27 − 0.25A + 0.0388B − 0.1563C − 0.09AB + 0.5AC + 0.4875BC − 0.6998A^2^ − 0.4073B^2^ − 2.17C^2^.

**Figure 2:**
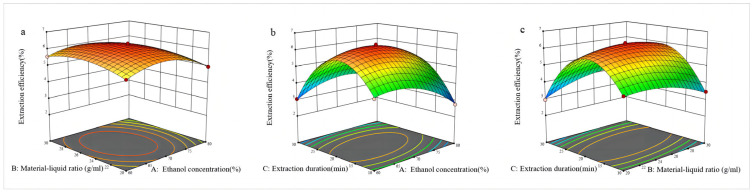
(**a**–**c**) Response surface plots illustrating the impact of ethanol concentration (X1: 60%, 70%, 80%), extraction time (X2: 10, 20, 30 min), and the ratio of the solvent to material (X3: 1:20, 1:25, 1:30 mL/g) on the extraction yield of GRS.

Based on the analysis from Design-Expert 13.0 software, the response surface can be obtained. From Figure 2a under the condition of a zero level of extraction time and with the solvent–material ratios remaining constant, the extraction rate initially increased and then decreased as the ethanol concentration increased. With the ethanol concentration remaining constant, the extraction rate of GRS also exhibited a trend of initially increasing and then decreasing as the solvent–material ratio continuously increased. Figure 2b indicates that under the condition of a zero level of the material–solvent ratio, the change in the response surface plot was significant, indicating that ethanol concentration and extraction time had a more significant impact on the extraction rate of GRS. The extraction rate of GRS initially increased and then decreased with rising ethanol concentration. Similarly, with longer extraction times, the extraction rate of GRS exhibited a more pronounced trend of initially increasing and then decreasing. This indicates that extraction time has a greater impact on the extraction rate of GRS than ethanol concentration. Figure 2c shows that when ethanol concentration was at the zero level, and the extraction time was maintained at a constant value, the extraction rate initially increased and then decreased with the increase in the material–solvent ratio, but the magnitude of the change was not significant. With the material–solvent ratio fixed, as the extraction time continuously increased, the extraction rate exhibited a trend of initially increasing and then decreasing, and the magnitude of change was significant.

According to the equation fitted by the software, the optimal extraction process for GRS was determined to be an ethanol concentration of 67.992%, a solvent–material ratio of 1:25.188 mL/g, and an extraction time of 19.454 min. The predicted extraction rate of GRS under these conditions was 6.302%. Considering the convenience in practical application, the optimal extraction process was revised to an ethanol concentration of 68%, a solvent–material ratio of 1:25 mL/g, and an extraction time of 20 min.

### 2.3. Chemical Component Analysis of Total Flavonoids

**Figure 3:**
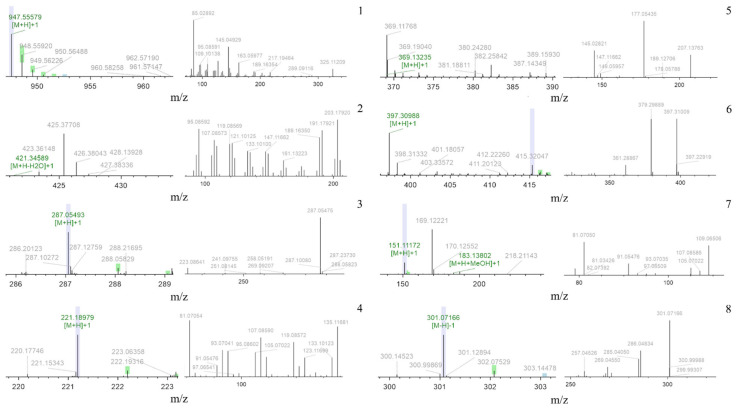
MS and MS/MS spectra profiles of 8 constituents in GRS measured by LC-MS.

Total saponins were characterized by LC-MS, and their chemical constituents were compared with different databases, including mzCloud, mzVault, and ChemSpider, for characterization. Additionally, to screen for the most abundant components, compounds with peak areas greater than 1 × 108 were analyzed. The retention time (Rt), [M–H]-, MS/MS [M–H]-, [M+H]-, MS/MS [M+H]-, calculated mass, and formula for each component are listed in Table 1. The primary and secondary mass spectra of each group are shown in Figure 3. Fragment ions of compounds **1** and **2** were compared with previously reported data [26,27], with ions at m/z 947.6 and 425.4 matching ginseng saponin Rd and oleanolic acid, respectively. Similarly, fragment ions appearing at m/z 287.1 and 221.2 may correspond to kaempferol [28] and caryophyllene [29], respectively. Additionally, fragment ions observed at m/z 163.1 and 397.3 are considered characteristic fragments of curcumin and Shuyuzaogan (dioscin) (compounds **5** and **6**) [30,31]. The ions observed at m/z 169.1 and 301.1 are attributed to carvone (compound **7**) [32] and hesperetin (compound **8**) [33], respectively. Among these eight compounds, oleanolic acid, caryophyllene, Shuyuzaogan (dioscin), and ginseng saponine Rd belong to the terpenoid compounds, while curcumin and kaempferol belong to the polyphenolic compounds, and the remaining two belong to the flavonoid compounds.

### 2.4. Immunomodulatory Analysis of Mice

#### 2.4.1. Effect of Ginseng Fibrous Root Extract on Peripheral Blood Hematology in Mice

**Figure 4:**
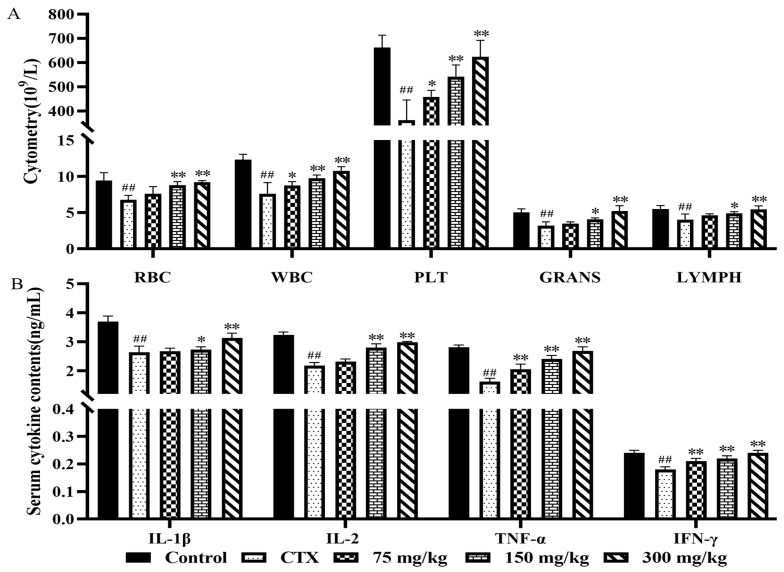
Effects of GRS on blood cells and cytokines in immunosuppressed mice. (**A**) Effect of ginseng fibrous root extract on peripheral blood hematology in mice; (**B**) effect of ginseng fibrous root extract on serum cytokine levels in mice. ## *p* < 0.01, compared with Control group; * *p* < 0.05, ** *p* < 0.01, compared with CTX group.

During the process of the cellular immune response, the interaction of peripheral blood cells plays a crucial role, ensuring the health of the body by detecting and isolating foreign pathogens and other abnormal substances. As shown in Figure 4A, in the model group of mice, RBC, WBC, PLT, GRANS, and LYMPH were significantly decreased (*p* < 0.01). Compared with the model group, the low-dose group showed significant increases in WBC and PLT (*p* < 0.05); the medium-dose group showed extremely significant increases in RBC, WBC, and PLT (*p* < 0.01), and significant increases in GRANS and LYMPH (*p* < 0.05); and the high-dose group showed extremely significant increases in RBC, WBC, PLT, GRANS, and LYMPH (*p* < 0.01). These results indicate that a ginseng fibrous root extract could restore white blood cell, lymphocyte, and neutrophil counts, and hematopoietic function. This method has also been used by Kseniya Bushmeleva to study the immunomodulatory effects of Aronia melanocarpa Extract, demonstrating that the extract promotes rapid recovery of the rat immune system, normalizing white blood cell counts, and enhancing monocyte and neutrophil phagocytic function [34,35].

#### 2.4.2. Effect of Ginseng Fibrous Root Extract on Serum Cytokine Levels in Mice

Inflammation is an integral part of the immune response; thus, immunomodulatory effects are of considerable significance in this regard [36]. Interferon-gamma (IFN-γ) is a lymphokine with broad immunomodulatory effects. As shown in Figure 4B, the serum levels of cytokines IL-1β, IL-2, TNF-α, and IFN-γ in the model group of mice were extremely significantly decreased (*p* < 0.01). In the low-dose group, serum levels of TNF-α and IFN-γ were extremely significantly higher than those in the model group (*p* < 0.01), while there were no significant differences in serum IL-1β and IL-2 levels (*p* > 0.05). In the medium-dose group, the serum level of the immune factor IL-1β was significantly increased (*p* < 0.05), while the serum levels of cytokines IL-2, TNF-α, and IFN-γ were extremely significantly increased (*p* < 0.01). In the high-dose group, the serum levels of cytokines IL-1β, IL-2, TNF-α, and IFN-γ in mice were extremely significantly increased (*p* < 0.01). Ginseng saponin and ginseng extracts exhibit clear anti-inflammatory effects, reducing the production of inflammatory molecules in vitro as well as levels of inflammatory cytokines and messenger RNA (mRNA) expression [37,38,39]. For instance, they reduce the secretion of cytokines IL-1β, IL-6, and TNF-α, and inhibit the translocation of NF-κB by avoiding the phosphorylation of inhibitor κB (IκBα) [40,41,42]. Additionally, the American ginseng soft branch significantly improved CD4+CD8-T lymphocyte counts, the splenic index, and serum IgA, IgG, TNF-α, and IFN-γ levels by modulating the ERK/MAPK pathway [43].

### 2.5. In Vitro Antioxidant Activity Results Analysis

DPPH is a highly stable nitrogen-centered free radical, and its ability to be cleared is observed when it encounters nitrogen atom inhibitors, resulting in a lighter color when measured by ultraviolet spectrophotometry. This characteristic, along with its repeatability, makes it widely used in antioxidant assays. As shown in Table 2, GRS exhibited scavenging ability against DPPH free radicals, IC_50_ = 0.893 mg/mL. When GRS reaches its maximum scavenging capacity of 92.1%, the concentration is 10 mg/mL. ABTS+ turns blue-green after oxidation and is commonly used in the laboratory to assess the antioxidant capacity of substances due to its repeatability. Therefore, it is also widely used in antioxidant assays. GRS shows increased scavenging ability against ABTS+ free radicals with increasing concentration, IC_50_ = 0.210 mg/mL. Thus, GRS exhibited good scavenging ability against ABTS+, with a maximum clearance rate of 98.7% observed at a concentration of 1.6 mg/mL, demonstrating excellent antioxidant activity. In this experiment, the scavenging ability against DPPH and ABTS+ was tested to study its in vitro antioxidant activity. Although GRS is not as effective as VC, we have demonstrated that GRS at certain concentrations has a strong scavenging ability, indicating antioxidant activity. Compared with other plant extracts, GRS exhibits a significantly lower IC_50_ value of 0.893 mg/mL for scavenging DPPH free radicals, surpassing the IC_50_ value of 6.95 mg/mL for saponins extracted from caryophyllene [44]. Regarding an ABTS antioxidant capacity analysis, GRS has an IC_50_ value of 0.210 mg/mL for scavenging ABTS free radicals, which is notably superior to the IC_50_ value of 0.53 mg/mL for a crude Veratrum nigrum extract [45] in scavenging ABTS free radicals.

## 3. Materials and Methods

### 3.1. Reagents and Laboratory Animals

Ginseng fibrous roots were selected from the fibrous roots of ginseng grown in the authentic medicinal forests of Huanren Manchu Autonomous County, Liaoning Province, China (latitude 40°54′~41°32′ N, longitude 124°27′~125°40′ E). The medicinal materials were identified by Professor Fu Junfan from the College of Life Engineering, Shenyang Institute of Technology. After being washed and naturally dried, the ginseng fibrous roots were ground into fine powder for further use.

A ginseng saponin Re standard (batch number B21055-20 mg, HPLC ≥ 98%) was purchased from Shanghai yuanye Bio-Technology Co., Ltd. (Shanghai, China);Vitamin C (ascorbic acid), 2,2′-azino-bis (3-ethyl benzothiazoline-6-sulfonic acid) diammonium salt (ABTS), 1,1-Diphenyl-2-picrylhydrazyl (DPPH)and potassium persulfate were all purchased from Beijing Solarbio Science & Technology Co., Ltd. (Beijing, China); Mouse interleukin-2 (IL-2), interferon-γ (IFN-γ), mouse interleukin-1β (IL-1β), and tumor necrosis factor-α (TNF-α) assay kits were purchased from Beijing Solarbio Science & Technology Co., Ltd. (Beijing, China); Cyclophosphamide (batch number: C849559-25 mg) was purchased from Shanghai Macklin Biochemical Technology Co., Ltd. (Shanghai, China); Other reagents were purchased from Sinopharm Chemical Reagent Co., Ltd. (Shenyang, China).

Fifty Kunming SPF male mice, weighing 18–22 g, were purchased from Liaoning Changsheng Biotechnology Co., Ltd. (Benxi, China), with the experimental animal license number SCXK (Liao) 2023-0001. The mice were acclimated for at least one week in a room maintained at 24 degrees Celsius, pathogen-free, with a 12/12 h light–dark cycle. These mice were fed standard laboratory food and drank freely. Animal experiments were carried out according to the international regulations on the use and welfare of experimental animals, and have been approved by the Ethics Committee of Shenyang Institute of Technology (SITLLBA2023102, approval date: 10 July 2023).

### 3.2. Experimental Design

#### 3.2.1. Single-Factor Experiments

In this experiment, single-factor experiments were conducted to investigate the effects of three main factors: the ethanol solution concentration, ultrasonic extraction time, and material–solvent ratio. Response surface design was then used to determine the optimal extraction conditions, with the extraction yield of total saponins from ginseng fibrous roots as the indicator. The effects of ethanol solution concentrations ranging from 50% to 90%, ultrasonic extraction times ranging from 10 to 30 min, and material–solvent ratios ranging from 1:10 to 1:30 were investigated. The extraction was performed twice. When evaluating any one influencing factor, the third level of the other two factors was selected.

#### 3.2.2. Determination of Content of Total Saponins

In this experiment, the total saponin content of ginseng fibrous roots was determined using the spectrophotometric method [46]. Firstly, a standard curve for ginseng saponin Re was established. The regression equation of the standard curve was Y = 11.309X + 0.0393 (R^2^ = 0.9907). Precisely weighed 2 mg of ginseng saponin Re was dissolved in methanol and made up to 2 mL. Aliquots of 0.03 mL, 0.06 mL, 0.09 mL, 0.12 mL, 0.15 mL, and 0.18 mL of the control solution were taken and added to separate 1.5 mL centrifuge tubes. The solvent was evaporated using a water bath, followed by the addition of 0.2 mL of a freshly prepared 5% vanillin–acetic acid solution and 0.8 mL of perchloric acid. The tubes were shaken well and then heated in a constant-temperature water bath at 60 °C for 15 min and immediately cooled in running water for 2 min. The solution was transferred to 10 mL centrifuge tubes, and 5 mL of acetic acid was added, followed by thorough mixing. Acetic acid was used as a blank control. The absorbance was measured at 556 nm using ultraviolet spectrophotometry.

#### 3.2.3. Extraction Process of Total Saponins of Ginseng Fibrous Roots

The experiment referenced the ultrasonic extraction process described by Zhang et al. [47] to extract GRS. Specifically, 5 g of ginseng fibrous root powder was accurately weighed and placed in a 250 mL conical flask. Different concentrations of ethanol were added according to the corresponding solvent–material ratios. Ultrasonic treatment was performed at a power of 330 W for varying durations. After ultrasonication, the extraction solution was filtered by vacuum filtration, and the filtrate was evaporated under normal pressure. Subsequently, 15 mL of distilled water was added to dissolve the extract in a 50 mL centrifuge tube. Then, defatting was conducted twice using 15 mL of ether. After centrifugation at 4000 r/min for 90 s, the lower layer of the solution was collected. The defatting process was repeated once more with 15 mL of ether, and the lower layer of the solution was collected again. A water-saturated n-butanol solution was then added for extraction, and the n-butanol solution was collected. Finally, the solvent was evaporated under normal pressure to obtain GRS.

#### 3.2.4. Optimization of Extraction Conditions by Box–Behnken Design

Through single-factor experiments, three levels of each factor were determined. Then, a Box–Behnken design (BBD) with three variables and three levels was introduced to optimize the extraction parameters. The effects of three factors, the ethanol concentration (X1), extraction time (X2), and solvent–material ratio (X3), on the extraction efficiency of total saponins of ginseng fibrous roots were investigated, with the total saponin extraction rate of ginseng fibrous roots as the evaluation index. Different levels of the three factors were examined, as shown in Table 3. When considering the influence of each factor on the total saponin content of ginseng fibrous roots, the other two factors were set at the intermediate level, with each set of three runs conducted in parallel.

### 3.3. Component Analysis

#### 3.3.1. Sample Handling

The ginseng fibrous root extract powder was weighed to approximately 100 mg and added to 1 mL of 80% methanol, followed by vortex mixing. A total of 2–3 Zirconium dioxide grinding beads were added, and the mixture was ground for 5 min and then vortexed for 10 min. The mixture was centrifuged at 4 °C for 10 min at a centrifugal force of 20,000× *g*. The supernatant was filtered through a 0.22 μm filter membrane, and the filtrate was analyzed by a machine.

#### 3.3.2. Detection Conditions

Mass spectrometry conditions—Ion source: Electrospray ionization (ESI); Scan mode: Switching between positive and negative ion scans; Detection mode: Full mass/dd-MS2; Resolution: 70,000 (full mass) and 17,500 (dd-MS2); Scan range: 100.0~1500.0 m/z; Spray voltage: 3.2 kV (positive); Capillary temperature: 300 °C; Collision gas: High-purity argon (purity ≥ 99.999%); Sheath gas: Nitrogen (purity ≥ 99.999%), 40 Arb; Auxiliary gas (Aux gas heater temperature): Nitrogen (purity ≥ 99.999%), 15 Arb, 350 °C; Data acquisition time: 30.0 min.

Chromatographic conditions—Column: AQ-C18 150 × 2.1 mm 1.8 μm, Welch; Flow rate: 0.30 mL/min; Aqueous phase: 0.1% formic acid/water solution; Organic phase: Methanol; Column temperature: 35 °C; Autosampler temperature: 10.0 °C; Autosampler injection volume: 5.00 μL; Gradient elution (elution conditions in Table 4).

### 3.4. Immunomodulation

Fifty male mice were acclimated to the housing conditions. They were then randomly divided into 5 groups, with each group containing 10 mice. These groups included a blank control group, an immune suppression model group, and low-, medium-, and high-dose groups (75, 150, 300 mg/kg). For the first 3 days of the experiment, the blank control group received intraperitoneal injections of physiological saline, while the other groups received intraperitoneal injections of cyclophosphamide at a dose of 80 mg/kg. From the 4th to the 17th day of the experiment, the blank control group and the model group were orally gavaged with double distilled water, while the high-, medium-, and low-dose groups were orally administered ginseng fibrous root extracts. On the 18th day, blood samples were collected from the mice, and the serum was obtained by centrifugation at 3000 r/min for 10 min. An enzyme-linked immunosorbent assay (ELISA) was used to detect the levels of cytokines (IL-2, IFN-γ, TNF-α, IL-1β) in the mouse serum, following the instructions provided with the assay kits. Plasma samples from each group of mice were collected in tubes containing an EDTA anticoagulant. Subsequently, appropriate amounts of plasma were aspirated using QBC tubes inserted into pipettes, followed by centrifugation for 5 min at 12,000 r/min using an IDEXX dry hematology analyzer for detection.

### 3.5. In Vitro Antioxidant Activity

#### 3.5.1. Evaluation of DPPH Free Radical Scavenging Activity

The DPPH assay was conducted following the method described in reference [48] with certain modifications. Different concentrations of sample solutions (1 mL) (0.625 mg/mL, 1.25 mg/mL, 2.5 mg/mL, 5 mg/mL, 10 mg/mL) were mixed with 500 μL of the DPPH solution and thoroughly blended. The mixture was then allowed to react at room temperature in the dark for 30 min. Absorbance values at 517 nm were recorded, and each sample was tested in triplicate. A vitamin C (VC) solution was used as a positive control, and 50% ethanol served as the blank control. DPPH scavenging rates at various concentration levels were calculated according to Formula (1).

(1) $$DPPH\;radical\;scavenging\;rate=(1-\frac{A_{1}}{A_{0}})\times 100\%$$

where A_1_ represents the absorbance at 517 nm for the sample group, while A_0_ represents the absorbance at 517 nm for the blank control group.

#### 3.5.2. Evaluation of ABTS Free Radical Scavenging Activity

The ABTS assay was conducted following the method described in reference [49] with certain modifications. Sample solutions of different concentrations (0.1 mg/mL, 0.2 mg/mL, 0.4 mg/mL, 0.8 mg/mL, 1.6 mg/mL) (500 μL) were mixed with 500 μL of the ABTS solution and thoroughly blended. The mixture was then allowed to react at room temperature in the dark for 30 min. The absorbance at 734 nm was measured, and each sample was tested in triplicate. The VC solution was used as the positive control, and 50% ethanol served as the blank control. The ABTS scavenging rates at various concentration levels were calculated according to Formula (2).

(2) $$ABTS\;radical\;cation\;scavenging\;rate=\left(1-\frac{A_{1}}{A_{0}}\right)\times 100\%$$

where A_1_ represents the absorbance at 734 nm for the sample group, while A_0_ represents the absorbance at 734 nm for the blank control group.

## 4. Conclusions

The ginseng fibrous root, as a medicinal herb with both food and therapeutic properties, is widely favored by people in Asia, and its research and development are gradually deepening. In this study, ultrasound-assisted extraction was employed to extract saponin compounds from ginseng fibrous roots, and their chemical constituents were identified, along with investigating their immunomodulatory activity. Through single-factor experiments and response surface optimization, the total flavonoid extraction rate reached 6.34%. The optimal process was achieved with an ethanol concentration of 68%, a solvent–material ratio of 1:25 mL/g, and an extraction time of 20 min, which proved to be feasible. The obtained total ginseng saponins effectively alleviated cyclophosphamide-induced immunosuppression in mice, demonstrating good immunomodulatory activity and free radical scavenging ability. In this study, Q-Orbitrap high-resolution liquid chromatography–mass spectrometry (LC-MS) technology was applied, which features high resolution and sensitivity in separation, enabling a rapid identification of chemical constituents in ginseng fibrous root extracts. Qualitative identification was achieved by comparing the information of reference compounds in databases and differences in retention time. Eight compounds were identified from the ginseng fibrous root extract, including oleanolic acid, caryophyllene, Shuyuzaogan (dioscin), ginseng saponin Rd, curcumin, kaempferol, carvone, and hesperetin. The results of this study provide theoretical support for the utilization of ginseng fibrous roots in the research and development of antioxidant cosmetics and free radical scavenging products, as well as immune-enhancing health care products and other related products.

## Figures and Tables

**Table 1:** Identification results of chemical constituents of total saponins from fibrous roots.

No.	Rt (min)	[M–H]-	MS/MS [M–H]-	[M+H]-	MS/MS [M+H]-	Calculated Mass	Formula	Proposed Molecule	Reference
1	19.00	—	—	947.6	85.1, 145.1, 163.1	946.5	C_48_ H_82_ O_18_	Ginsenoside Rd	[26]
2	18.33	—	—	425.4	95.1, 191.2, 203.2	438.3	C_30_ H_48_ O_3_	Oleanolic acid	[27]
3	11.99	—	—	287.1	223.1, 288.1	286.1	C_15_ H_10_ O_6_	Kaempferol	[28]
4	14.21	—	—	221.2	81.1, 107.1, 135.1	220.2	C_15_ H_24_ O	Caryophyllene oxide	[29]
5	13.31	—	—	163.1	145.0, 177.1, 207.1	368.1	C_21_ H_20_ O_6_	Curcumin	[30]
6	12.61	—	—	397.3	361.2, 379.3	396.3	C_27_ H_42_ O_3_	Diosgenin	[31]
7	10.71	—	—	169.1	81.1, 91.1, 109.1	150.1	C_10_ H_14_ O	Carvone	[32]
8	13.18	301.1	285.0, 286.1, 301.1	—	—	302.1	C_16_ H_14_ O_6_	Hesperetin	[33]

**Table 2:** Results of the antioxidant activities of GRS extracts.

Indicators	Antioxidants	Equation of Equations	R^2^ of Linear Fit	IC_50_
DPPH	GRS	y = 42.027x + 52.053	0.991	0.893 (mg/mL)
	VC	y = 73.395x + 75.315	0.9912	0.319 (μg/mL)
ABTS+	GRS	y = 58.206x + 89.486	0.9908	0.210 (mg/mL)
	VC	y = 73.395x + 75.315	0.9951	0.99 (μg/mL)

**Table 3:** Independent variables and their levels in Box–Behnken design for GRS.

Levels	Ethanol Concentration (X 1)/(%)	Extraction Time (X 2)/(min)	Ratio of Solvent to Material (X 3)/(mL/g)
−1	60	10	20
0	70	20	25
1	80	30	30

**Table 4:** Chromatographic gradient elution.

Time (min)	Aqueous Phase (%)	Organic Phase (%)
1	98	2
5	80	20
10	50	50
15	20	80
20	5	95
27	5	95
28	98	2
30	98	2

## Data Availability

The original contributions presented in the study are included in the article; further inquiries can be directed to the corresponding authors.

## Supplementary Materials

The following supporting information can be downloaded at: https://www.mdpi.com/article/10.3390/molecules29122770/s1, Table S1: Box–Behnken design with experimental results.
