# Supplementary material for: Free Radical Scavenging Effect and Immunomodulatory Activity of Total Saponins Extract of Ginseng Fibrous Roots
Source: Molecules. 2024 Jun 11;29(12):2770. doi: 10.3390/molecules29122770 (PMC11206437; doi:10.3390/molecules29122770)
Supplement: Supplementary file 1 [file molecules-29-02770-s001.zip › molecules-3029941-supplementary.pdf]

Table S1 Box-Behnken design with experimental results

Test number	*A* ethanol concentration/%	*B* material-liquid ratio/(mL•g^{-1})	*C* extraction time /min	*Y* extraction rate /%
1	0	0	0	6.23
2	-1	0	-1	4.25
3	1	1	0	4.85
4	0	1	1	4.02
5	1	-1	0	4.97
6	1	0	1	3.56
7	0	0	0	6.24
8	-1	0	1	3.05
9	-1	-1	0	5.30
10	0	0	0	6.23
11	0	0	0	6.34
12	-1	1	0	5.54
13	0	-1	1	2.95
14	1	0	-1	2.76
15	0	-1	-1	4.35
16	0	1	-1	3.47
17	0	0	0	6.32
